# Personal

**Published:** 1889-02

**Authors:** 


### Personal.

S. Weir Mitchell, M. D., LL.D., has been elected Professor of Diseases of the Mind and Nervous System in the Philadelphia Polyclinic and College for Graduates, an additional chair upon that subject having been created. Dr. Mitchell is widely known as an author and teacher, and the Polyclinic is to be congratulated upon securing his services in the capacity named.

---

Dr. William A. Hammond, Surgeon-General United States Army (retired), has again taken up his residence in Washington, where he has built a commodious Sanitarium for diseases of the nervous system. This beautiful structure is a model private hospital in all its appointments, and was opened on the sixth of January, 1889, with half its rooms already filled with patients. Dr. Hammond's well-known and well-earned reputation for skill in his special department of medicine, is a guarantee that the inmates of his Sanitarium will receive every attention and care that modern medical science can provide.

---

Announcement.—E. B. Treat, publisher, 771 Broadway, New York, will publish, early in 1888 the seventh annual issue of the English "Medical Annual," a résumé, in dictionary form, of new remedies and new treatment that have come to the knowledge of the medical profession throughout the world during 1888. In its enlarged and widened sphere it will take the name of "The International Medical Annual," and will be published in one octavo volume of about 600 pages, at $2.75, under copyright protection, and issued simultaneously in London and New York.

